# The Early Indicators of Functional Decrease in Mild Cognitive Impairment

**DOI:** 10.3389/fnagi.2016.00193

**Published:** 2016-08-12

**Authors:** Alexandre Kubicki, Lilian Fautrelle, Julien Bourrelier, Olivier Rouaud, France Mourey

**Affiliations:** ^1^Unité 1093, Cognition, Action et Plasticité Sensorimotrice, Institut National de la Santé et de la Recherche MédicaleDijon, France; ^2^Université de Bourgogne Franche Comté, Unité de Formation et de Recherche (UFR) Sciences et Techniques des Activités Physiques et Sportives (STAPS)Dijon, France; ^3^Institut de Formation aux Métiers de la Santé (IFMS), Nord Franche-Comté, Hôpital Nord Franche-ComtéMontbéliard, France; ^4^Université Paris Ouest Nanterre La Défense, Unité de Formation et de Recherche (UFR) Sciences et Techniques des Activités Physiques et Sportives (STAPS)Nanterre, France; ^5^Centre de Recherche sur le Sport et le Mouvement, CeRSM, Unité de Formation et de Recherche (UFR) Sciences et Techniques des Activités Physiques et Sportives (STAPS)Nanterre, France; ^6^Centre Mémoire Ressources et Recherche, Centres Hospitaliers Universitaires (CHU), Dijon-BourgogneDijon, France; ^7^Unité de Formation et de Recherche (UFR), Santé, Université de Bourgogne Franche ComtéDijon, France

**Keywords:** Mild Cognitive Impairments, muscle synergy, Anticipatory Postural Adjustments, motor control, cognitive functions

## Abstract

**Objectives:** Motor deficiency is associated with cognitive frailty in patients with Mild Cognitive Impairments (MCI). In this study we aimed to test the integrity in muscle synergies involved in an arm-pointing movement in functionally unimpaired MCI patients. We hypothesized that early motor indicators exist in this population at a preclinical level.

**Methods:** Electromyographic signals were collected for 11 muscles in 3 groups: Young Adults (YA), Older Adults (OA), and MCI patients. The OA and MCI groups presented the same functional status. Each subject performed 20 arm-pointing movements from a standing position.

**Results:** The main differences were (1) an earlier activation of the left Obliquus internus in MCI compared with OA group, (2) an earlier activation for the MCI compared with both OA and YA. The temporal differences in muscle synergies between MCI and OA groups were linked with executive functions of MCI patients, assessed by the trail making test. Moreover, the results show a delayed activation of the right Biceps Femoris and the right Erector Spinae at l3 in MCI and OA compared with YA.

**Interpretation:** The motor program changes highlighted in our patient MCI group suggest that discrete modifications of the motor command seem to exist even in the absence of functional impairment. Instead of showing an indication of delayed muscle activation in the MCI patients, our results highlight some early activation of several trunk muscles.

## Background

Mild Cognitive Impairments (MCI) is an intermediate stage between typical age-related cognitive changes and dementia. There is growing evidence in the literature about the association of sensory-motor dysfunction with cognitive frailty in aging (Albers et al., 2015). Several research teams focused on the broad correlation between motor and cognitive capacities in aging. In patients with MCI, there is a solid body of evidence supporting the motor-related changes. These studies have reported some gait and balance deficiencies (Boyle et al., 2005; Aggarwal et al., 2006; Verghese et al., 2008; Gras et al., 2015), which become even more significant in dual-task conditions (Montero-Odasso et al., 2014), and a greater fall rate in this population (Doi et al., 2014). Interestingly, a value of the gait speed test that predicts evolution toward cognitive frailty has been also highlighted (Buracchio et al., 2010).

These studies assessed overall motor functions (i.e., functional abilities) in MCI patients. Motor function involves the peripheral musculoskeletal system (information capture and torque production), the peripheral nervous system (information conduction) and the central nervous system (CNS; motor planning and programming) (Caffarra et al., 2010; Cisek and Kalaska, 2010). As the symptoms of MCI are mainly cognitive, and thus related to central modifications, it could be interesting to identify potential impairments affecting this central part of motor behavior in MCI patients.

To assess the integrity of this central part of motor control, researchers have usually focused on the preparatory period, which includes motor program processes, and well-known as Anticipatory Postural Adjustments (APA; Belen'kii et al., 1967; Massion, 1992; Desmurget and Grafton, 2000; Maloney and Mamassian, 2009). Indeed, our CNS has the ability to coordinate posture and movement efficiently, especially by means of feed-forward processes that allow the anticipated recruitment of several muscles before the beginning of any self-generated perturbation of our balanced-system (Massion, 1992). These APA are mainly involved in maintaining the integrity of the balance function and can be challenged in several situations (Horak, 2006). Actually, anticipatory activations of trunk muscles, such as transversus abdominis and multifidus, were found to be delayed in chronic low-back-pain patients (Hodges and Richardson, 1996). In the same vein, acute and experimentally-induced pain seems to shift the anticipation activations from a deep (transversus abdominis and internal obliquus) to a superficial muscular layer (external obliquus) (Moseley and Hodges, 2005).

Normal aging also challenges these APA (Man'kovskii et al., 1980; Inglin and Woollacott, 1988; Rogers et al., 1992; Woollacott and Manchester, 1993; Bleuse et al., 2006; Kanekar and Aruin, 2014), with greater severity in frail elderly adults (Kubicki et al., 2012a). Neurological disorders such as stroke or Parkinson's disease also impair the ability of the CNS to produce efficient APA (Latash et al., 1995; Garland et al., 1997; Elble and Leffler, 2000).

Nonetheless, there are very few studies about APA in cognitively impaired patients in the literature. Elble and Leffler reported that postural and focal reaction times were slower in Alzheimer Disease (AD) patients, but without specific impairment in the postural preparatory period, than in age-matched controls without AD (Elble and Leffler, 2000). To our knowledge, there is no information in the literature about APA in MCI patients.

This study is warranted by the lack of data about this important question, especially when considering the link between APA and balance function (Robinovitch et al., 2013). Our aim was to test APA integrity in MCI patients, without functional impairments, during an arm raising task. Our hypothesis was that MCI patients would have delayed anticipation activations compared with age-matched controls, but that the global synergy of muscle activations would be respected. To better understand these potential changes, and to take into account the APA impairment highlighted in normal aging, we included a group of young adults (YA) in our experiment.

## Materials and methods

### Participants

Participants (*n* = 42) were divided into 3 groups: MCI (Mild Cognitive Impairment group); OA (Older Adults group), and YA (Young Adults group). All the participants were right handed as assessed by the Edinburgh Handedness Inventory (Oldfield, 1971).

MCI subjects (*n* = 14) were included at the Memory, Resources and Research Centre (CMRR) of Dijon University Hospital, France, during their annual medical consultation. Inclusion criteria were (1) a recent diagnosis of amnestic MCI according to the criteria of NIA-OA (Albert et al., 2011), (2) an absence of a diagnosis of dementia due to Alzheimer's disease, (3) to be aged 60 years or over, (4) no other neurological disease, such as Parkinsonism syndrome or pyramidal deficiency, (5) no musculoskeletal deficiency that caused pain, balance deficit or restricted function. To be more precise, the Alzheimer's disease and the Parkinsonism syndrome were clinically diagnosed by an experienced neurologist. The Alzheimer's disease was diagnosed following the “recommendations from the National Institute on Aging-Alzheimer's Association workgroups on diagnostic guidelines for Alzheimer's disease” (Albert et al., 2011), and the Parkinsonism syndrome following the recommendations from the “MDS clinical diagnostic criteria for Parkinson's disease” (Postuma et al., 2015). One more time, both were retained as exclusion criteria. These subjects were recruited during their annual medical consultation, during which they provided their written consent to participate in the study.

MCI was diagnosed by an experienced neurologist (OR) by means of the criteria of NIA-OA (Albert et al., 2011). A few patients underwent associated tests, such as MRI T2 flair to detect hyper-signals (Wahlund et al., 2001). Their neuropsychological assessment included the following tests for the executive and attentional functions: the Trail Making Test (TMT A and TMT B) (Reitan, 1958; Corrigan and Hinkeldey, 1987; Gaudino et al., 1995; Lezak et al., 2004) and the Digit Span (Forward and Backward). The Free Cued Selective Reminding Test (FCSRT) and the Delayed Matching to Sample (DMS 48) were done to assess their memory functions. At inclusion, the mean scores were 4.85 (Digit Span Forward); 3.71 (Digit Span Backward); 46.81 s (TMT A); 128 s (TMT B); 18.6 (FCSRT RL, on 48); 34.5 (FCSRT RT, on 48); 92.8 % (DMS 48, Set 1); 90.7 (DMS 48, Set 2).

OA subjects (*n* = 14) were volunteers who had no previous experience as subjects in experimental research. These individuals had no medical diagnosis of or self-assessed cognitive deficiency, such as memory loss or executive problems, and met the same (3-4-5) inclusion criteria as MCI patients (see above).

YA subjects (*n* = 14) were recruited at the Sport Sciences Department of the University of Dijon (Burgundy University, France). They were recruited with respect to the (4-5) inclusion criteria, they had no medical diagnosis of or self-assessed cognitive deficiency, and were aged between 20 and 35 years.

This work has been carried out in accordance with The Code of Ethics of the World Medical Association (Declaration of Helsinki) for experiments involving humans. The privacy rights of human subjects have been observed at all times.

### Experimental device and procedure

As a prerequisite, the gait speed test was done at the beginning of the session to test the functional ability of each patient. All participants performed a short warm-up of the shoulder muscles of 1 min duration consisting of global circumduction movements prior to the start of the experiment. They were instructed to copy the same movements of the experimenter who performed these movements in front of the participants.

**Figure 1:**
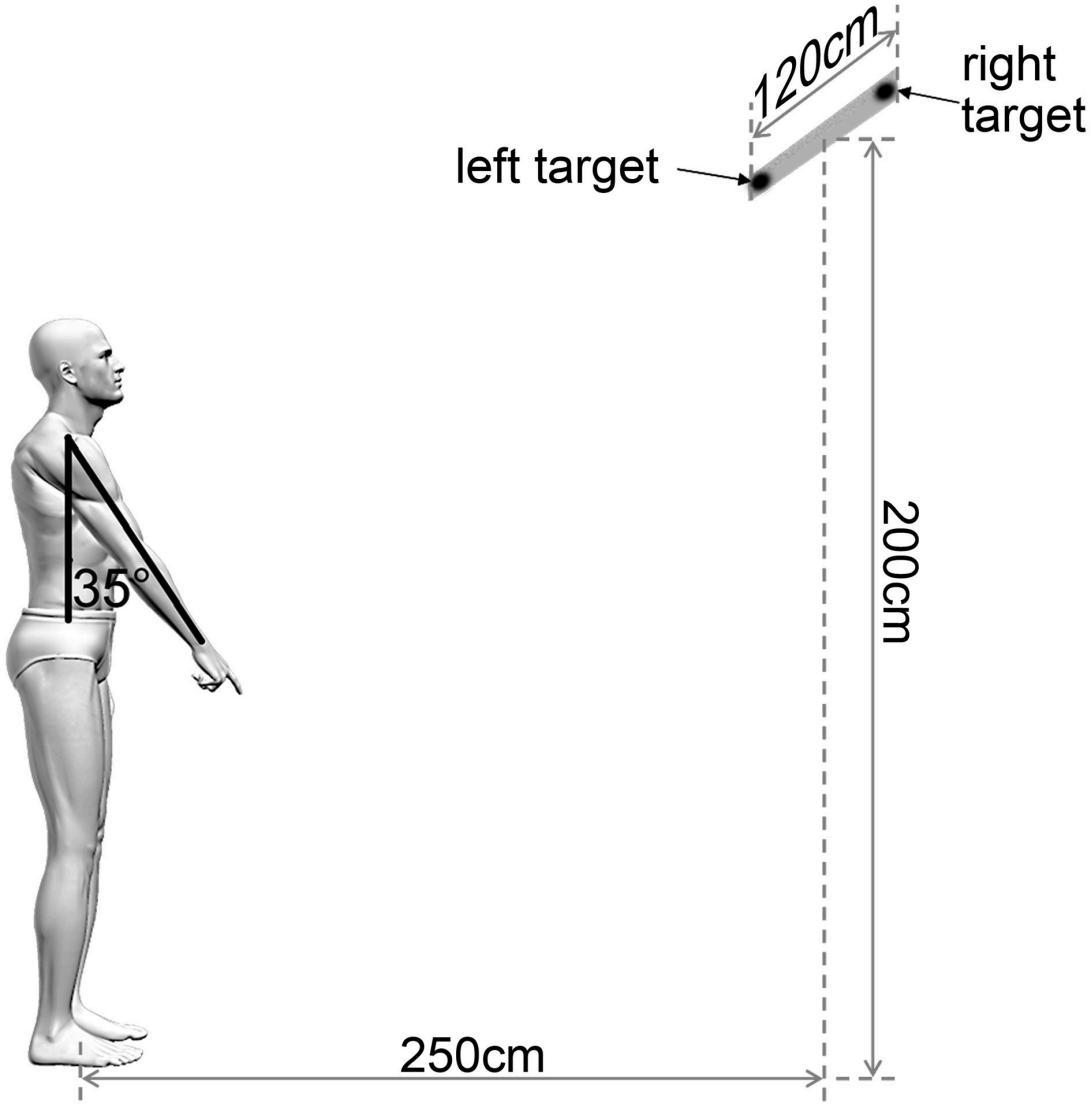
**Experimental arm raising task**. View of the experimental set-up for the arm raising task showing a participant in initial position and the two possible targets. The central point of the bar between the two targets was situated exactly in front of the participants' right shoulder. Participants were asked to point their index finger at the target (left or right) which was lit intermittently.

The starting position was the following: Participants stood upright on the floor (feet were oriented at 15° on both sides of the sagittal plane, with 15 cm between the two medial malleoli), the left arm was positioned in alignment with the trunk and the right index finger pointing toward the ground, with an angle of 35° between the arm and trunk (Figure 1). The subjects were required to keep their eyes fixed on a horizontal bar placed at 2 m from the floor and 2.5 m from the participants' feet. Two diodes, 120 cm apart, were fixed on this horizontal bar. The central point between the two diodes was situated exactly in front of the participants' right shoulder. Participants were told to point their index finger at the diode (left or right) which was switched on intermittently.

Participants were unaware of the location (left or right) of the visual stimuli. They were asked to raise their arm as fast as possible and to start as quickly as possible after the appearance of the visual stimuli. This complex reaction time task, with a relative uncertainty about the target location (and thus the motor program to initiate), made it possible to generate greater APA than a simple reaction time task (Slijper et al., 2009; Kubicki et al., 2012b). They were told to point to the diode, to keep their arm raised for a few seconds, and then lower their arm and move their index finger back to the initial starting position. The participants performed 20 pointing trials. All the included participants were able to understand these instructions, to complete the warm-up sequence and the 20 reaching movements.

### Participant equipment

Two systems were used to accurately measure (i) the movements performed and (ii) timing of muscle activations during these movements.

- Right upper limb kinematics were recorded using the Vicon® system (Oxford metrics group, UK). Participants wore 5 motion sensors on the following anatomical sites: nail of the index finger; dorsal aspect of the scaphoid bone; lateral aspect of the elbow (lateral epicondyle); anterior aspect of the shoulder (acromion).
- Surface Electromyographic (EMG) activity of 11 muscles was collected. According to the EMG literature concerning the detection of APA (Ng et al., 1998; Moseley and Hodges, 2005), we focused our measurements on the lower limbs and trunk muscles in reference to the main muscle involved in arm-pointing: the Anterior Deltoid (Crenna and Frigo, 1991). See **Figure 4C** displaying the electrodes placement.

EMG was recorded on both sides of each subject (right = r, left = l) for the rectus femoris (RF), biceps femoris (BF), obliquus internus (OI), erector spinae at the third lumbar vertebra (ESl3), erector spinae at the seventh dorsal vertebra (ESd7), and on the right side for the anterior portion of the deltoidus (DA). To determine the positioning of the surface electrodes, the participants were instructed how to selectively activate each recorded muscle individually (Hislop and Montgomery, 2009). These electrodes were placed parallel to the muscle fibers with an interelectrode distance of 2.4 cm.

### Data recording and statistical analysis

Gait speed (m.s^−1^) was collected on a 4.5 m distance according to Fried et al. (2001). All EMG signals were preamplified at the source (VICON®, Oxford Metrics Group) and were recorded at a frequency of 2000 Hz. Raw EMG signals were first bandpass filtered between 5 and 400 Hz and then full-wave rectified and filtered using a no-lag averaging moving-window algorithm (window size: 10 ms). As previously defined by Fautrelle et al. (2010), in this study, we determined the initiation time as the delay between illumination of the diode (the “go-signal”) and the beginning of significant muscle activation (Fautrelle et al., 2010). To do this for the 11 recorded muscles, the EMG values after the illumination of the diode and the EMG baselines were compared for each value using *t*-tests for each muscle in each group. The EMG baselines were computed as the mean integrated activity of each muscle from −2 to −1 s before the first diode was lit and when the participants maintained the initial position. The first instant at which the *P*-value was lower than 0.05 for a minimum duration of 50 ms determined the beginning of muscle activation necessary to perform the pointing movements.

We chose to calculate the timing of each muscle activation with reference to activation of the Anterior Deltoid and we named this parameter the activation timing. Thus, the activation timing of muscles involving in the APA had negative values and positive values refer to post-deltoid contractions. This EMG synergy allowed us to clearly identify the muscles involved principally in APA in each group, without confounding factors associated with the electro-mechanical delays (Zhou et al., 1995). A last aim of this study was to investigate some potential relationships between muscle activation timings (i.e., physiological parameters from motor control) and the results of clinical tests in MCI group. In this way, the “absolute difference score” (ADS) in milliseconds (ms) were calculated between MCI and OA participants for the muscles showing significant differences in their activation timings between MCI and OA groups only. Finally, a cumulative score of these differences (Total ADS) was calculated by summing the 2 ADS scores in absolute value.

### Statistical analysis

Concerning activation timings, in order to determine potential differences between groups, we first checked that each variable was normally distributed (Shapiro–Wilk *W*-test, all the *p*-values are > 0.1) and had equivalent variances (Levene's test, all the *p*-values are > 0.2). Outliers detected using extreme studentized deviate tests (ESD tests; Rosner, 1983) were removed. For more precision, these outliers were due to rare and furtive loss of contact between EMG sensors and elderly frail skin (24/280 for MCI, 8/280 for OA groups) or excessive sudation (30/280 for YA group, with a maximum of 7/20 for one participant due to excessive sudation). Then a one-way ANOVA was conducted in each recording muscle while the experimental group (x3: YA, OA, MCI) remained the categorical factor (i.e. the independent variable).

Concerning muscular activation rates which were reported in percentage for each participant, Shapiro–Wilk *W*-tests showed non-normal (but log-normal) distributions for the 10 tested muscles. Consequently, transformations were performed (Bartlett, 1947) before investigate the potential differences in the muscular activation rates using 10 different one-way ANOVA, similarly to the analyses of the activation timings.

*Post-hoc* analyses were done with Scheffe tests when necessary. For all these statistical treatments, the significance level was set at *p* < 0.05. Moreover, according to Cohen (Bartlett, 1947), the effect size was specified by the partial eta squared (η_*p*_^2^), and a value ≥ 0.14 was considered as a large effect, ≥ 0.6 as a medium effect, and ≥ 0.1 as a small effect (Cohen, 1988; Sink and Stroh, 2006).

Concerning some potential relationships between muscle activation timings and the results of well-known clinical tests in MCI group, a multiple regression model was applied to the Total ADS (as a dependent variable) with the following explanatory (independent) variables: Mini-Mental State score (MMS), Walhund score, Gait speed score, Part A and Part B of the Trail Making Test scores (TMT A and TMT B). According to Cohen (1988) and Sink and Stroh (2006), the effect size of the amount of variance accounted for was specified by the *R* squared (*R*^2^) score. A *R*^2^ ≥ 0.14 was considered as a large effect.

## Results

### Participants characteristics

Participants' characteristics are summarized in Table 1. Women accounted for 35.71, 64.28, and 57.14% in the YA, OA, and MCI groups, respectively. The MCI and OA groups were no different for age (*p* = 0.353) and maximal velocity of the index movement (*p* = 0.069). Consistent with previous data showing the slow-down of movement in aging (Ketcham et al., 2002), there was a significant difference between aged participants of both the MCI and OA groups and the YA group concerning the index maximal velocity [*F*_(2, 728)_ = 165.91, *p* < 0.001, η_*p*_^2^ = 0.89]. Moreover, the functional status of the MCI and OA were equivalent, as attested by the comparable Gait Velocity (*p* = 0.448).

**Table 1:** **Patient characteristics (Mean ± Standard Deviation) for each group: Young Adults (YA); Older Adults(OA); Mild Cognitive Impairment (MCI)**.

**Parameter**	**YA group**	**OA group**	**MCI group**	***p*-value for HY/OA**	***p*-value for OA/MCI**	***p*-value for HY/MCI**
Age (Years)	28.72 ± 5.65	70.62 ± 4.14	70.15 ± 7.17	< 0.001	0.353	< 0.001
Height (cm)	177 ± 3.67	166 ± 4.82	165 ± 4.23	0.112	0.487	0.214
Weight (Kg)	71 ± 5.48	79 ± 6.91	76 ± 7.33	0.081	0.192	0.154
Gait Speed (m.s^−1^)	1.11 ± 0.05	0.94 ± 0.11	0.91 ± 0.13	< 0.001	0.448	< 0.001
Index MV (m.s^−1^)	6.781 ± 1.58	4.889 ± 1.04	4.67 ± 1.46	< 0.001	0.069	< 0.001

*p-values are presented for between-group differences. LDS fisher-tests (post-hoc) were done on the Group effects showed by the ANOVA (see Material and Methods section for further details). “Index MV” means Index Maximal Velocity*.

Finally, The ANOVA showed a main Group effect for AD (anterior deltoid muscle) reaction time [*F*_(2, 728)_ = 9.367, *p* < 0.001, η_*p*_^2^ = 0.91]. The *post-hoc* decomposition showed that AD reaction time was significantly shorter in the YA group than in both the MCI and OA groups (*p* < 0.001) but there was no difference between the OA and MCI groups (*p* = 0.103).

### Muscle activation timing between groups

**Figure 2:**
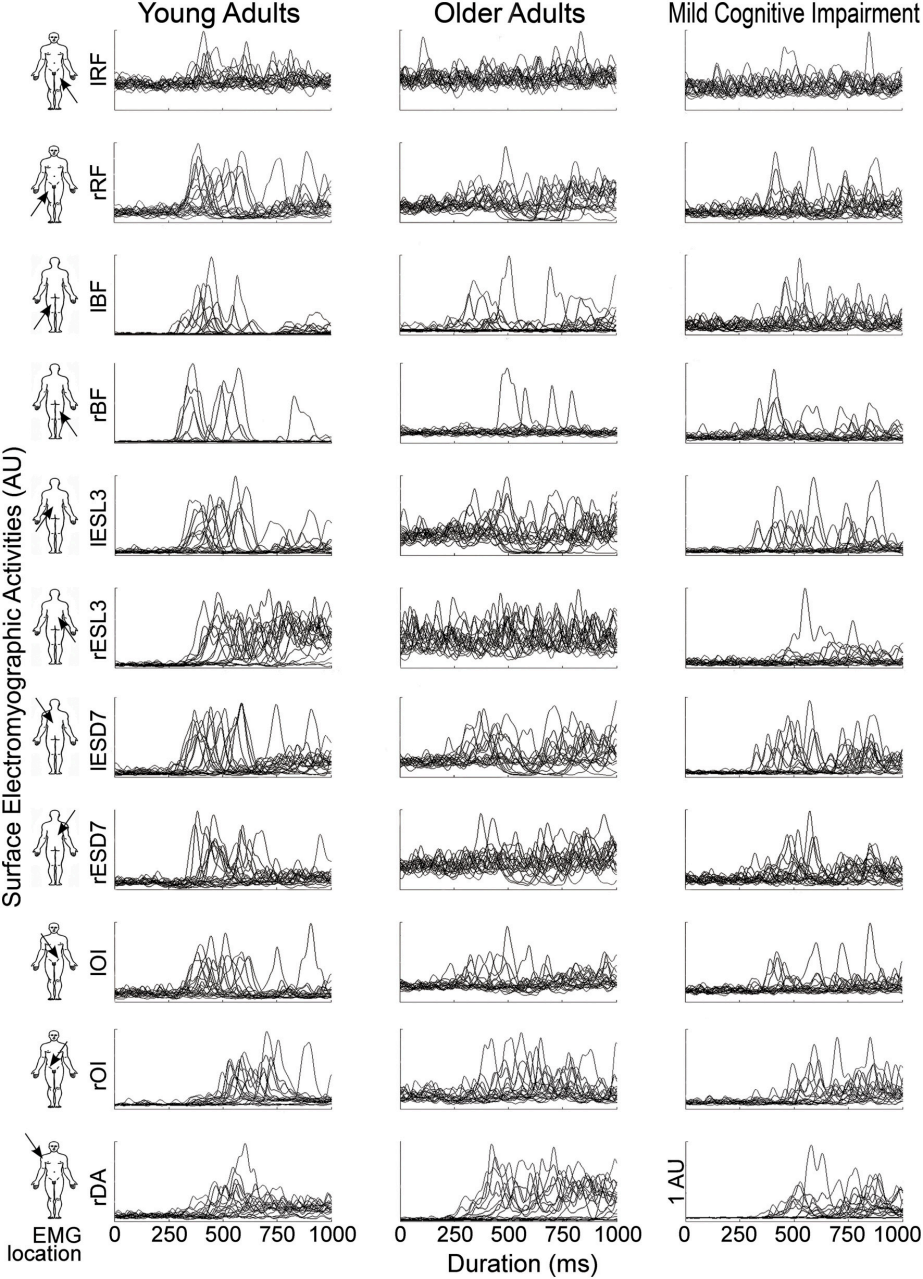
**EMG activities of the 11 recorded muscles (line) for one typical participant in each group (column), from left to right: Young Adults (YA); Older Adults(OA); Mild Cognitive Impairment (MCI)**. Each graph represents the EMG signals of every trials from the illumination of the diode (*t* = 0) until 1 s. The EMG activities (V) were bandpass filtered between 5 and 400 Hz and then full-wave rectified. For the sake of clarity in this figure, EMG signals were normalized by the maximum EMG value (V) for each muscle and for each participant and were represented in arbitrary units (AU).

To clarify the results and highlight the potential differences between groups, muscle synergies were studied, with data for YA subjects, considered optimal, as the reference. Figure 2 shows the raw EMG data obtained for 20 trials for a typical subject of each group.

**Figure 3:**
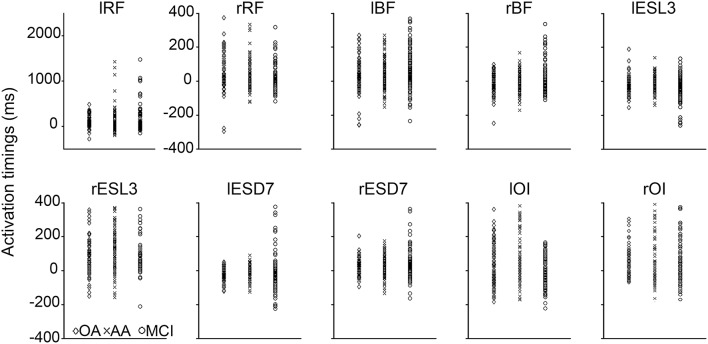
**Scatter plot of the activation timings raw data for all the trials of every participant in the three experimental groups**. These activation timings (ms) were computed for each tested muscle with reference to the anterior deltoid activation. The timing of muscles involving in the APA had negative values and positive values refer to post-deltoid contractions. In every graph, the activation timings of the young adults (YA) were reported in the left column (diamond markers), the older adults (OA) in the middle column (cross markers), and the mild cognitive impairment group (MCI) in the right column (circle markers).

Figure 3 shows the scatter of the overall dataset of the timing of muscle activations (all the trials of all participants in the three experimental groups).

In the YA group, 3 muscles participated in the APA in the following order: lESl3, lESd7, and rBF.

The ANOVA showed a main Group effect for several muscles: lESl3 [*F*_(2, 693)_ = 14.92, *p* < 0.001, η_*p*_^2^ = 0.15]; rBF [*F*_(2, 647)_ = 4.226, *p* = 0.015, η_*p*_^2^ = 0.14]; rRF [*F*_(2, 266)_ = 3.7367, *p* = 0.025, η_*p*_^2^ = 0.23]; rESl3 [*F*_(2, 383)_ = 9.2164, *p* < 0.001, η_*p*_^2^ = 0.22]; lOI [*F*_(2, 459)_ = 6.8433, *p* = 0.0012, η_*p*_^2^ = 0.44].

Comparing the timing of muscle activation between YA and OA groups, we found 1 significant difference: the activation timing of the rBF (*p* = 0.008), was delayed for the OA group compared with the YA group.

Interestingly, the *post-hoc* analysis revealed earlier activations of the lESl3 (*p* < 0.001) and the lOI (*p* < 0.05) in MCI compared with OA group. This chronological difference was also significant between MCI and YA groups for the lESl3 (*p* = 0.019).

Logically, activation timings of the MCI group were significantly delayed compared with the YA group for the rBF (*p* = 0.014), the lRF (*p* = 0.027), and rESl3 (*p* = 0.028).

**Figure 4:**
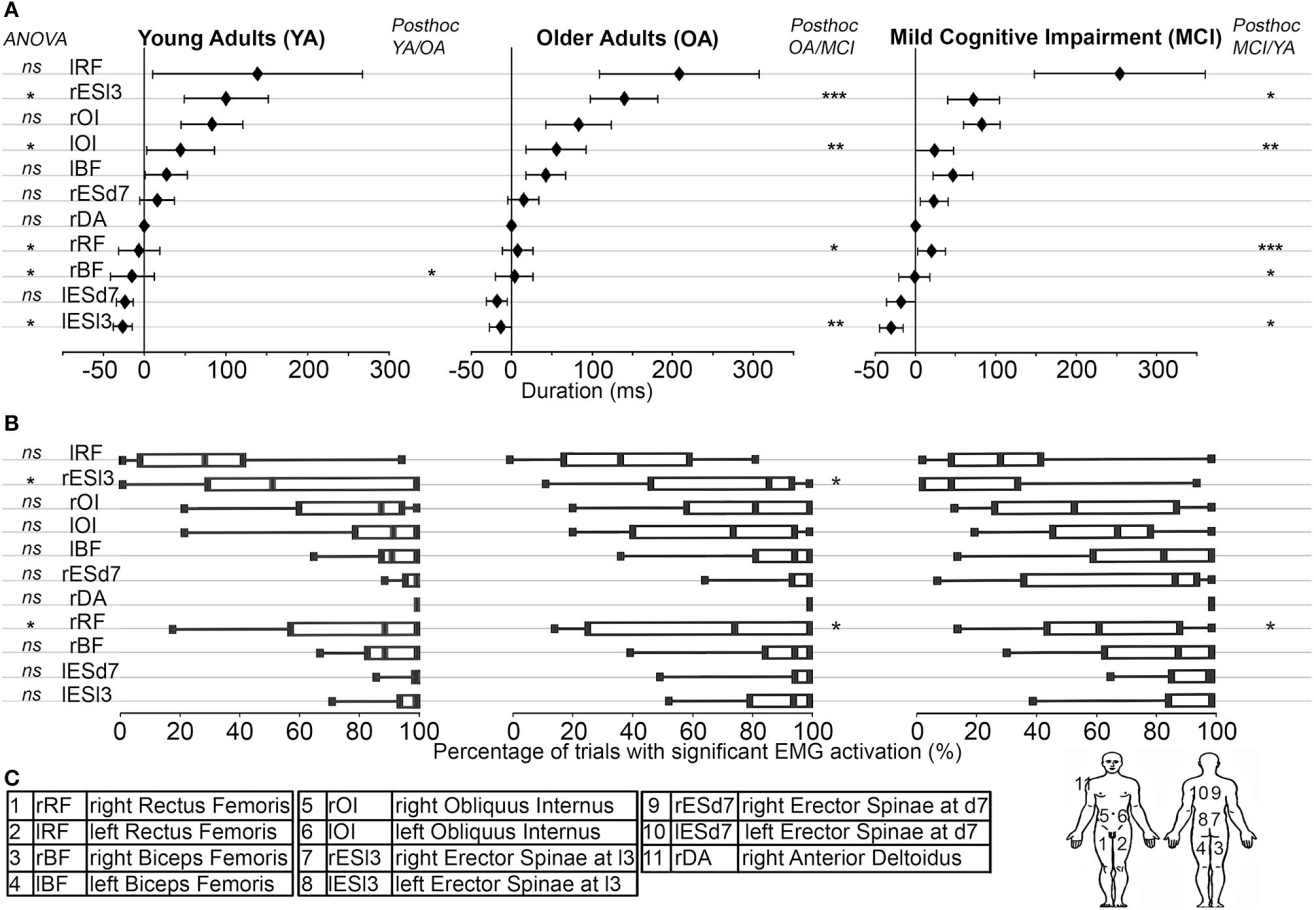
**(A)** Muscle synergy used in the pointing movement. From left to right: Young Adults (YA); Older Adults (OA); Mild Cognitive Impairment (MCI). On the y-axis, muscles were represented from the bottom up in the chronological order of their activations in the YA group. On the x-axis, timing of muscle activations (ms) were represented with reference to that of the Anterior Deltoid (Mean ± Standard Deviation). The ANOVA results were displayed on the left (ns = *p* > 0.05; ^*^p < 0.05). *Post-hoc* results were displayed (when necessary) from left to right for the YA/OA; the OA/MCI and the MCI/YA analysis (^*^p < 0.05; ^**^p < 0.01; ^***^p < 0.001). **(B)** Muscle activation rates: The percentages of trials with significant EMG activation were represented with box plots including median and quartiles, in the same layout than above. The ANOVA results were displayed on the left (ns = *p* > 0.05; ^*^p < 0.05). *Post-hoc* results were displayed (when necessary) from left to right for the YA/OA; the OA/MCI and the MCI/YA analysis (^*^p < 0.05). **(C)** For the sake of clarity, the muscle abbreviations, names and locations were reported here.

All the average muscle activations, with the ANOVA main effect and the *post-hoc* analysis between the three groups are presented on the Figure 4A.

### Muscle activation rates between groups

To determine the robustness of muscle synergy objectively, the activation rate for each muscle in each group was calculated. For each muscle, this rate corresponded to the percentage of trials showing significant muscle activation. Differences were found for only two muscles. The rESl3 [*F*_(2, 9489)_ = 13.51, *p* < 0.0001, η_*p*_^2^ = 0.92] and the rESD7 [*F*_(2, 2422)_ = 7.59, *p* = 0.001, η_*p*_^2^ = 0.89] were less frequently activated in the MCI group compared with the OA group (*p* < 0.001 and *p* = 0.002 respectively) and the YA group (*p* < 0.001 and *p* = 0.001 respectively). These results are shown in Figure 4B.

### Muscle activation timing and clinical data in the MCI group

**Figure 5:**
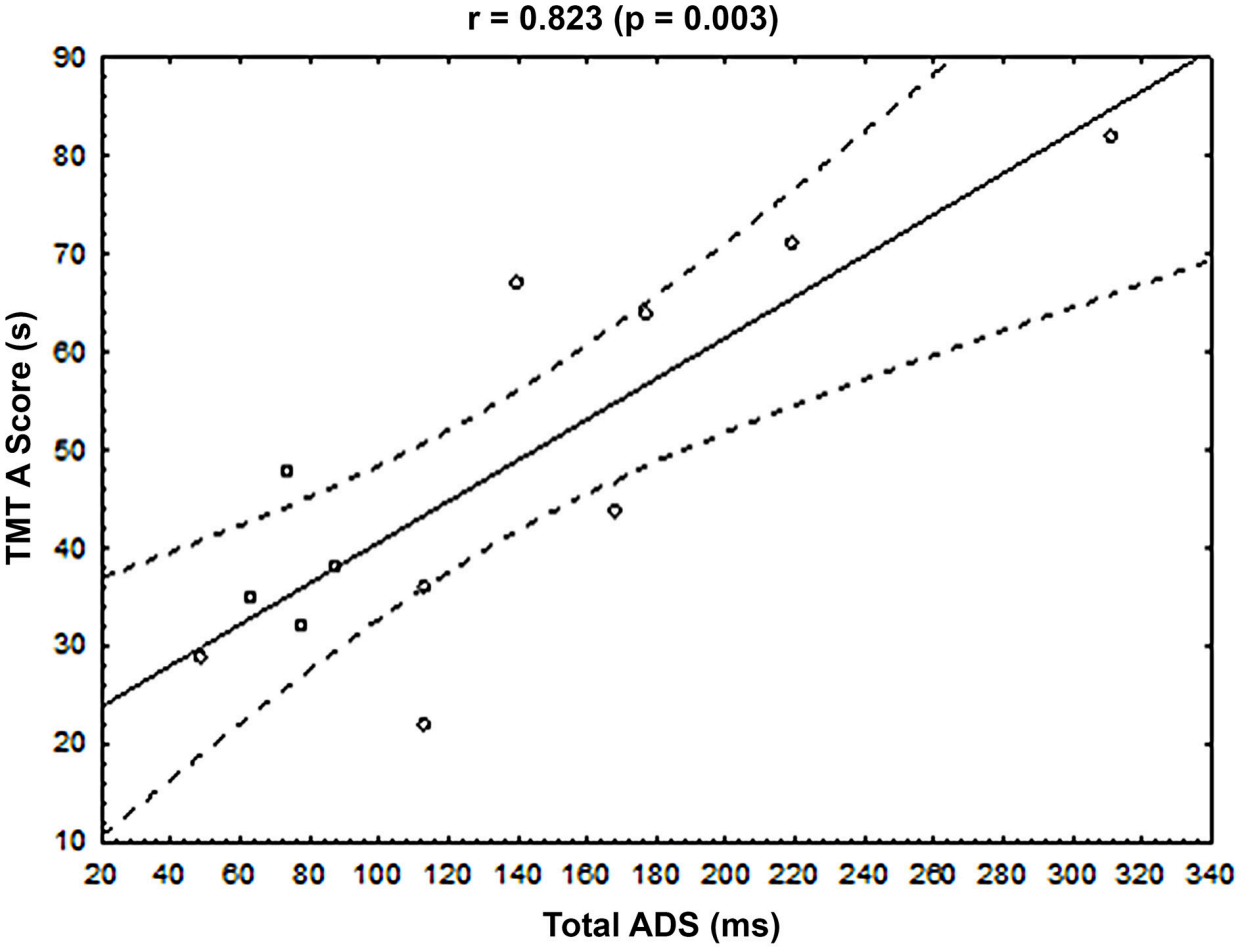
**Relationship between the cumulative Absolute Difference Score (Total ADS, x-axis, ms) and the Trail Making Test score, part A (TMT A score, y-axis, s) for each MCI patient**. The Pearson and associated *p*-value were displayed at the top of the graph. Black line represented the regression line, and dotted lines represented the 95% confident interval.

The ADS was calculated to highlight potential links between muscle activation timings and clinical data in MCI patients, in milliseconds (ms), between MCI and OA subjects, for each of the 2 muscles showing significant differences in their activation timing (see Section Muscle Activation Timing between Groups and Figure 4A): the lESl3 and the lOI. Please note here that the decision was made to exclude the lRF muscle from this analysis, as its timing was also modified in the MCI group compared to the OA group, because this muscle activation presented an important variability (see on Figure 3 lRF' graph, and Figure 4A) and a poor activation rate (see on Figure 4B). A cumulative score of these differences (Total ADS) was calculated by summing the 2 ADS scores in absolute value. A multiple regression model was applied to this Total ADS (as a dependent variable) with the following explanatory (independent) variables: Mini-Mental State score (MMS), Walhund score, Gait speed score, Part A and Part B of the Trail Making Test scores (TMT A and TMT B). The result of this complementary analysis is that the variance explained by the model was *R*^2^ = 0.819, with only one significant explanatory variable: The TMT A (Beta = 1.08; *p* = 0.03). To represent this relationship graphically, the Total ADS was plotted with the TMT A scores for all patients of the MCI group (see Figure 5).

## Discussion

The aim of this study was to measure differences in muscle synergies used by MCI patients during an arm-raising task, especially in the preparatory period of postural control (APA) associated with arm movement. In young adults (YA group), our results showed -EMG- APA sequences originating from 3 muscles (among the 10 postural muscles we assessed): two contralateral trunk muscles (lESl3 and lESD7) and one ipsilateral lower limb muscles (rBF), which was in accordance with the main literature about APA organization associated with arm movements (Cordo and Nashner, 1982; Friedli et al., 1984; Ketcham et al., 2002; Bonnetblanc et al., 2004; Bouisset and Do, 2008).

### Effects of aging on muscle synergy in arm raising

Our results clearly showed a delay of ipsilateral Biceps Femoris (right) in the older adults compared with the YA. This significant difference probably reflect the first impairment of APA in these active aged adults. This impairment of motor prediction mechanisms has been highlighted in the literature through several studies using EMG signals (Man'kovskii et al., 1980; Inglin and Woollacott, 1988; Rogers et al., 1992; Woollacott and Manchester, 1993; Bleuse et al., 2006; Kanekar and Aruin, 2014). In these studies, the authors showed several delays in the timing of muscles that participate in APA: postural muscles closer to the muscle directly involved in the movement were recruited. The worst case was described by Woollacott and Manchester (1993). In this study, postural muscle activation occurred after the arm movement had begun. Indeed, the authors showed a clear impairment of the APA produced by contralateral erector spinae muscles in normal aged adults, and a decreased activation rate for the ipsilateral quadriceps muscles. By contrast, in our study concerning the same aged population (69.53 ± 3.12 years), we only found a delay for one muscles (rBF), and no impairment in the recruitment rate of any muscle compared with our young subjects. It is essential to point out that in our study and contrary to the Woollacott study, the participants were instructed to point to the target as fast as possible; consequently, the older adults pointed more slowly than the YA, the APA differences were thus not attributable to aging processes alone, but also to the differences of inertial forces associated with these self-generated perturbations.

### Effects of MCI on muscle synergy in arm raising

This study highlighted two main results about muscle synergy in arm raising in MCI patients.

First, our results show clearly that the muscle recruitment in MCI subjects is no more delayed than those of the subjects belonging to the OA group. On the contrary, two muscle activations are programmed even earlier: the lESl3, which is the first muscle involved in the APA in the YA subjects, and the lOI, which was one of the last muscles activated in both YA and OA groups. Concerning the lESl3, it is interesting to note that this muscle recruitment, corresponding to the first muscle anticipation, occurred earlier in our patient group compared with both the control subjects participating to this study (YA and OA). The lOI muscle, mainly involved in trunk stability, is also recruited early by the MCI patients, compared with the OA subjects. We have to recall here that the only difference between these two groups was the presence (MCI) or the lack (OA) of cognitive dysfunction, mainly involving memory loss (amnestic MCI). The maximal velocity of the arm movement was no different between these two groups. Consequently, these results indicate that cognitive symptoms of MCI patients are accompanied by fine motor changes mainly expressed by an earlier activation of two trunk muscles. We interpreted these results as follows: superficial trunk muscles are probably recruited earlier to increase the postural steadiness of the trunk and therefore to be able to compensate for the balance perturbation caused by the arm movement. This kind of trunk compensation has already been highlighted in a study by Morris and Allison or by Moseley and Hodges for low-back-pain patients (Moseley and Hodges, 2005; Morris and Allison, 2006), and could be interpreted as compensatory behavior of over-protection, as an over-estimation of the self-generated perturbation that will be induced by the movement. Our results are in accordance with these studies. In the same manner, MCI patients seem to adopt a more cautious mode of motor control, adapting their feedforward control to better stabilize the locomotor system.

Secondly, it is important to note that MCI patients presented the same activation rates as the other groups for most muscles studied in our task (see Section Muscle Activation Rates between Groups and Figure 4B), except for the rESl3 and rESD7. Therefore, these two muscles activated after the deltoid contraction, were also less frequently activated. Concerning the rESl3, the OA and MCI subjects are in late compared with the YA group. However, only the MCI subjects show a less robust use of this muscle, maybe by adaptation of their early activation of lESl3. Except for these two muscles, the robustness of muscle synergy seemed to be preserved in MCI patients throughout all the trials. These small modifications in the motor command robustness are in accordance with the lack of motor function impairment in this sample of patients, shown by identical gait speeds and index velocities in the two groups of aged adults.

Taken together, these results strongly suggest that overall motor prediction ability is not impaired in MCI patients. Rather, the anticipated motor command is modified toward an early recruitment of trunk muscles, probably aimed to increase its steadiness in a more cautious motor behavior.

Interestingly, the multiple regression model applied to the Total ADS (see Section Muscle Activation Timing and Clinical Data in the MCI Group), representing the muscular timing differences between OA and MCI patients, highlighted a relationship with the Trail Making Test, part A (TMT A). In other words, the higher the cumulative differences in muscle recruitment in absolute value, the higher the TMT A score. An increased TMT A score reflects poor executive functions, mainly in the processing speed of information (Reitan, 1958; Corrigan and Hinkeldey, 1987; Gaudino et al., 1995; Lezak et al., 2004), and have to be assessed in MCI patients since an early impairment of these functions have been highlighted in AD patients (Amieva et al., 2008). Consequently, our results indicated that TMT A score reflects both an aspect of the cognitive independency (processing speed of information) and an aspect of the physical independency: the ability to accurately coordinate posture and movement during self-generated perturbations.

This work presents several limits. Indeed, the three groups did not point with the same maximal velocity. Both the OA and the MCI subjects pointed more slowly than YA subjects. However, we have to consider two main aspects: First, this difference did not rule out comparisons of APA sequences between OA and MCI subjects. The YA subjects were mainly studied to validate the optimal command organization in light of previous work. Secondly, one may wonder whether or not it may have been more interesting to collect different set pointing speeds from our participants in order to avoid this velocity difference. However, voluntarily slowing the reaching movement could also modify the associated APA sequence (Krakauer and Shadmehr, 2007). With the instruction used in this study (“Please point to the illuminated diode as fast as possible”), the movement was probably more ecological.

Moreover, it is important to note a potential confounding factor associated with subject gender in our results. Indeed, the male/female ratio was not the same in the three groups, with a male preponderance in the YA group. However, if a gender bias exists, it did not concern the comparison between the OA and the MCI participants, but only the comparison between the YHA group and the two groups of aged adults.

Given these various elements, we can speculate that the changes in the motor program could precede the beginning of the motor-function impairment highlighted in the literature for the MCI population. These early motor indicators could be tested as others biomarkers in order to better predict the evolution of the dementia (Petersen et al., 2013). Further studies will be done to explore this hypothesis. In this vein, the included MCI patients will be followed longitudinally, in order to answer, at least in part, this interesting question.

## Author contributions

AK, Conception and design, Collection and assembly of data, Analysis and interpretation of the data, Drafting of the article, Final approval of the article, Critical revision of the article for important intellectual content, Statistical expertise. LF, Conception and design, Collection and assembly of data, Analysis and interpretation of the data, Drafting of the article, Final approval of the article, Critical revision of the article for important intellectual content, Statistical expertise. JB, Collection and assembly of data, Critical revision of the article for important intellectual content, Final approval of the article. OR, Provision of study materials or patients, Critical revision of the article for important intellectual content, Final approval of the article. FM, Conception and design, Analysis and interpretation of the data, Critical revision of the article for important intellectual content, Administrative, technical, or logistic support, Final approval of the article.

## Funding

This work was supported by the ANR MAAMI project (Maladie d'Alzheimer et Apprentissage Moteur Implicite). Research National Agency (ANR), France.

### Conflict of interest statement

The authors declare that the research was conducted in the absence of any commercial or financial relationships that could be construed as a potential conflict of interest.
